# Critical role of non-coding RNA-mediated ferroptosis in urologic malignancies

**DOI:** 10.3389/fimmu.2024.1486229

**Published:** 2024-10-31

**Authors:** Wei Li, Liying Zheng, Peiyue Luo, Tao Chen, Jun Zou, Qi Chen, Le Cheng, Lifeng Gan, Fangtao Zhang, Biao Qian

**Affiliations:** ^1^ The First Clinical College, Gannan Medical University, Ganzhou, Jiangxi, China; ^2^ Department of Urology, The First Affiliated hospital of Gannan Medical University, Ganzhou, Jiangxi, China; ^3^ Key Laboratory of Urology and Andrology of Ganzhou, Ganzhou, Jiangxi, China; ^4^ Department of Graduate, The First Affiliated Hospital of Gannan Medical University, Jiangxi, Jiangxi, China

**Keywords:** urologic malignancy, ferroptosis, miRNA, lncRNA, circRNA, molecular mechanism

## Abstract

Urologic malignancies, characterized by their high aggressiveness and metastatic potential, pose a significant public health challenge globally. Ferroptosis, a novel mode of cell death, typically arises from intracellular iron ion overload and the accumulation of lipid peroxides. This process has been shown to play a crucial regulatory role in various pathological conditions, particularly in cancer, including urologic cancers. However, the comprehensive regulatory mechanisms underlying ferroptosis remain poorly understood, which somewhat limits its broader application in cancer therapy. Non-coding RNAs (ncRNAs), which encompass microRNAs (miRNAs), long non-coding RNAs (lncRNAs), and circular RNAs (circRNAs), are non-coding transcripts that play pivotal roles in various physiological processes, such as proliferation, differentiation, apoptosis, and cell cycle regulation, by modulating the expression of target genes. The biological functions and potential regulatory mechanisms of ncRNAs in the context of cancer-related ferroptosis have been partially elucidated. Research indicates that ncRNAs can influence the progression of urologic cancers by affecting cell proliferation, migration, and drug resistance through the regulation of ferroptosis. Consequently, this review aims to clarify the functions and mechanisms of the ncRNA-ferroptosis axis in urologic cancers and to evaluate the clinical significance of ferroptosis-related ncRNAs, thereby providing new insights into cancer biology and therapeutic strategies that may ultimately benefit a diverse range of cancer patients.

## 1 Introduction

Urologic malignancies, including renal cancer, bladder cancer, and prostate cancer, have emerged as critical global public health concerns, with both incidence and mortality rates on the rise. According to the GLOBOCAN 2022 data report, the number of newly diagnosed urologic tumor cases increased from approximately 2.4 million in 2020 to 2.667 million in 2022, accounting for 13.35% of the total global cancer cases (1, 2). Global deaths attributed to urologic malignancies rose from 767,208 to 825,953, corresponding to an 8.4% increase in the share of all cancer-related deaths (1, 2). Prostate cancer ranks as the second most common cancer among men worldwide, following lung cancer, and is the fifth leading cause of cancer-related deaths (1). Bladder cancer, the second most prevalent urogenital malignancy, was the tenth most common cancer globally in 2020 (1, 2). Despite its relatively lower global incidence rate, renal cancer (including renal pelvis cancer) accounted for approximately 553,000 new cases and 208,953 deaths in 2022, underscoring its significance as a major type of urologic cancer (1). Given the trends of global population growth and aging, the incidence of urologic cancers is projected to rise further, posing significant challenges to public health and imposing considerable economic burdens on healthcare systems (3–5).

In recent years, significant advancements have been made in the diagnosis and treatment of diseases affecting the urinary system. However, urologic tumors, particularly those in advanced stages or with metastases, continue to be the leading cause of mortality among affected patients. For individuals with early-stage disease, radical surgery remains the preferred treatment option; nevertheless, challenges in preventing tumor metastasis and recurrence persist. In patients with advanced, unresectable tumors, prognosis is often poor due to their high resistance to chemotherapy and radiotherapy. Therefore, a comprehensive exploration of the molecular mechanisms underlying the initiation and progression of urologic tumors is essential for enhancing prevention efforts and optimizing clinical management strategies.

Ferroptosis, a novel form of iron-dependent programmed cell death, is characterized by its reliance on iron and the accumulation of lipid peroxides, which ultimately lead to cellular membrane rupture and cell death (6). Since its introduction by Dr. Brent R. Stockwell and colleagues in 2012, in their efforts to develop drugs targeting RAS proto-oncogene GTPase (RAS)-mutated cancer cells, dysregulation of the ferroptosis regulatory system has been linked to various physiological and pathological conditions (7, 8). Increasing evidence underscores the significant role of ferroptosis in multiple cancers, including urologic malignancies, thereby positioning it as a potential therapeutic target for these diseases (9–11). In contrast, non-coding RNAs (ncRNAs) are RNA molecules that do not directly participate in protein synthesis. Although they do not encode proteins, ncRNAs perform essential regulatory functions within cells and are involved in various biological processes, including development, proliferation, transcription, post-transcriptional modification, apoptosis, and cellular metabolism (12). NcRNAs are primarily categorized into three types: circular RNAs (circRNAs), long non-coding RNAs (lncRNAs), and microRNAs (miRNAs) (13). These ncRNA molecules exhibit tissue- and disease-specific expression patterns, indicating their potential as biomarkers for disease assessment (14). Recent studies have uncovered abnormal expression patterns of ncRNAs in various diseases, particularly in cancers, including urologic malignancies (15–18). In some instances, ncRNAs influence tumor progression by modulating ferroptosis pathways, highlighting their potential as therapeutic targets for regulating these pathways in cancer cells and thus providing new opportunities to enhance patient prognosis (19–22).

In summary, this review aims to explore the critical roles of ncRNAs and ferroptosis in the progression of urologic malignancies and their potential utility as biomarkers and targets for therapeutic intervention. By systematically evaluating and integrating recent research findings in related fields, this review aims to advance the understanding of the complex roles of ncRNAs in regulating ferroptosis-related pathways. Ultimately, this review aims to provide novel insights and strategies for the prevention, diagnosis, and treatment of urologic malignancies.

## 2 The current status of ferroptosis regulation mechanisms

**Figure 1:**
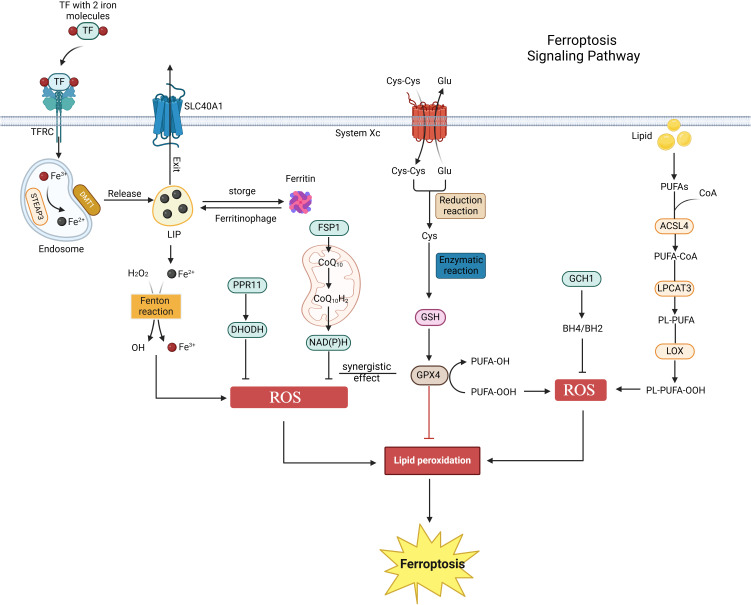
Ferroptosis signaling pathway. Ferroptosis is primarily regulated by iron metabolism, amino acid metabolism, and lipid metabolism. Initially, extracellular iron ions bind to TF/TFRC, subsequently entering the cytoplasm to form endosomes. These ions then enter the labile iron pool (LIP) via the actions of STEAP3 metal reductase and DMT1. Ferritin regulates the content of the LIP through autophagy or storage, whereas excess iron ions are expelled from cells via SLC40A1. The Fenton reaction involving Fe²^+^ and hydrogen peroxide generates reactive oxygen species (ROS), which promote ferroptosis. Secondly, cystine enters the cell via System Xc^-^ and is converted to glutathione (GSH) through reduction and enzymatic reactions. The GSH-GPX4 axis functions as an antioxidant defense system *in vivo*, inhibiting ferroptosis. Lastly, polyunsaturated fatty acids (PUFA) in lipids are enzymatically converted into PL-PUFA-OOH, promoting ferroptosis. Additionally, the FSP1-CoQ10-NAD(P)H, PPR11-DHODH, and GCH1-BH4 pathways also regulate ferroptosis.

Programmed cell death (PCD) is a complex and finely regulated biological process. It represents an active mechanism of cell death governed by genetic factors, intricately linked to the maintenance of organismal homeostasis and the onset of disease. PCD encompasses several distinct forms, including apoptosis, pyroptosis, necrosis, autophagy, and ferroptosis (23, 24). Ferroptosis, a unique form of cell death, was first identified in 2003 during experiments designed to screen small molecules for selective tumor cell killing and was formally designated by Stockwell et al. in 2012 (7, 25). Its core mechanism involves the disruption of intracellular redox homeostasis, particularly the depletion of glutathione (GSH) and the reduction of glutathione peroxidase 4 (GPX4) activity. This disruption impairs GPX4’s ability to effectively metabolize lipid peroxides, thereby triggering Fe²^+^-mediated lipid peroxidation reactions. This cascade ultimately leads to increased levels of reactive oxygen species (ROS), which are essential for the induction of ferroptosis (26). As a distinct mode of cell death, ferroptosis holds substantial scientific value in cell biology research and therapeutic applications. Further investigation into its mechanisms promises to provide new insights into prevention and treatment strategies for related diseases (**Figure 1**).

### 2.1 Iron metabolism and ferroptosis

A strong connection exists between iron metabolism homeostasis and ferroptosis. Within cells, hydrogen peroxide (H_2_O_2_) and iron ions (Fe²^+^) react through the Fenton reaction under acidic conditions, generating hydroxyl radicals (OH^-^), which are among the most destructive oxidants in reactive oxygen species (ROS) (27). These highly reactive ROS can initiate a series of oxidative reactions, including lipid peroxidation, DNA damage, and protein oxidation, ultimately leading to cell death (28). In the plasma, iron enters cells as Fe³^+^, bound to transferrin and its receptor (TF/TFRC), and is subsequently reduced to Fe²^+^ by the action of STEAP3 metalloreductase (29). Excess Fe²^+^ is expelled from cells via solute carrier family 40 member 1 (SLC40A1) and is oxidized back to Fe³^+^ during this process (30). Iron ions (Fe²^+^/Fe³^+^), as primary inducers of ferroptosis, can promote ROS generation through both enzymatic and non-enzymatic reactions (31). Studies have demonstrated that iron chelators can reduce free iron levels, inhibit lipid peroxidation, and thus suppress ferroptosis (32). Consequently, the regulation of iron uptake, utilization, storage, and efflux in cells and tissues can significantly influence susceptibility to ferroptosis (33).

### 2.2 Amino acid metabolism and ferroptosis

The glutathione-GPX4 axis in amino acid metabolism plays a crucial role in maintaining cellular homeostasis. System Xc-, composed of the light chain SLC7A11 and the heavy chain SLC3A2, mediates the reverse transport of intracellular glutamate and extracellular cystine at a 1:1 ratio. In this process, cystine is reduced to cysteine, which is a vital precursor for the synthesis of GSH (25). GSH serves as a non-enzymatic antioxidant within cells, essential for protecting against oxidative stress damage. The glutathione peroxidase family (GPX) consists of a group of antioxidant enzymes widely distributed in mammals, primarily comprising eight members, namely GPX1 through GPX8 (34). A common characteristic of these enzymes is their ability to catalyze the conversion of organic hydroperoxides (ROOH) or H_2_O_2_ into water and corresponding alcohols, thereby effectively eliminating intracellular reactive oxygen species (ROS) or lipid peroxides and safeguarding cells from oxidative stress damage (34, 35). Although all members of the GPX family contribute to the anti-oxidative stress process, GPX4 plays an irreplaceable role in ferroptosis, a specific form of cell death. GPX4 utilizes GSH as a reducing agent to directly act on lipid hydroperoxides present in the cell membrane, transforming them into innocuous alcohols, thereby inhibiting lipid peroxidation and preserving the integrity of the cell membrane (36). Consequently, the glutathione-GPX4 axis represents a critical antioxidant defense system in the body. Classical ferroptosis inducers, such as Erastin and Ras-selective lethal small molecule 3 (RSL3), specifically target System Xc- and GPX4, respectively, to exert their effects (37–39). Erastin binds to SLC7A11, blocking its transport function, which leads to impaired cystine transport and reduced GSH production. This results in the accumulation of lipid peroxides, inducing ferroptosis (40). RSL3 forms a covalent bond with GPX4, rendering it inactive, disrupting the cellular redox balance, promoting lipid peroxidation, and inducing ferroptosis (41).

### 2.3 Lipid metabolism and ferroptosis

Furthermore, lipid metabolism is intricately linked to ferroptosis. Lipid peroxidation predominantly occurs in membrane phospholipids that are rich in polyunsaturated fatty acids (PUFAs) (42, 43). Specific PUFAs, particularly those found in phosphatidylethanolamine (PE) and phosphatidylcholine (PC), serve as principal targets in ferroptosis (7, 43). ROS or lipoxygenases (LOXs) oxidize non-toxic PUFAs (PL-PUFAs) into toxic lipid peroxides (PL-PUFA-OOH), thereby inducing ferroptosis (32). Key enzymes in the lipid metabolism pathway, such as ACSL4 and LPCAT3, play a crucial role in regulating cellular lipid composition and sensitivity to ferroptosis (42, 44, 45). ACSL4 catalyzes the condensation of free PUFA with coenzyme A (CoA) to form derivatives, which are subsequently esterified into membrane phospholipids by LPCAT3. This process facilitates the onset of ferroptosis (46, 47). Therefore, supplementing PL-PUFA or inhibiting enzymes such as ACSL4 and LPCAT3 can modulate cellular sensitivity to ferroptosis (48–50).

### 2.4 Other parallel pathways and ferroptosis

Moreover, recent studies have identified several parallel pathways involved in ferroptosis. FSP1 is a novel ferroptosis resistance gene that operates under conditions of GPX4 deficiency or RSL3 inhibition. It is recruited to the plasma membrane through N-myristoylation, functioning as an NAD(P)H-dependent reductase that converts coenzyme Q10 to ubiquinol-10, thereby preventing the accumulation of lipid peroxides (51). The FSP1-CoQ10-NAD(P)H pathway acts as an independent parallel system that collaborates with GPX4 and glutathione to inhibit phospholipid peroxidation and ferroptosis (52). Conversely, DHODH, an enzyme that catalyzes the conversion of dihydroorotic acid to orotic acid, operates parallel to mitochondrial GPX4, inhibiting ferroptosis of the mitochondrial inner membrane by reducing ubiquinone (CoQ) to panalcohol (CoQH) (53). Recent studies have demonstrated that the PRR11-DHODH axis plays a crucial role in regulating ferroptosis in tumor cells. Miao et al. reported that PRR11 maintains the stability of DHODH by inhibiting its polyubiquitination-mediated degradation, thereby driving ferroptosis and drug resistance in glioblastoma (54). Additionally, genome-wide activation screening has identified a group of genes that can counteract ferroptosis, including GCH1 and its metabolites BH4/BH2 (55). GCH1-expressing cells synthesize BH4/BH2 through lipid remodeling to prevent phospholipid depletion and inhibit ferroptosis, a mechanism that operates independently of the GPX4/glutathione system (55). In conclusion, the regulatory mechanisms of ferroptosis remain to be fully elucidated, and their potential impacts on pathophysiological processes, particularly in urologic malignancies, warrant further investigation.

## 3 The role of ferroptosis in urological cancers

In recent years, ferroptosis has garnered significant attention in the field of cancer research, primarily due to its unique mechanisms and morphological characteristics (56). As research has progressed, the relationship between ferroptosis and malignant urinary system tumors has increasingly become a focal point of investigation. Relevant studies from the past year are summarized in **Table 1**. In urologic malignancies, various cancer-related signaling pathways regulate ferroptosis in tumor cells (57, 58). For instance, the phosphatidylinositol 3-kinase (PI3K)-AKT signaling pathway influences cell migration and invasion. In renal cell carcinoma, abnormal activation of this pathway promotes cancer cell migration and invasion, thereby accelerating tumor metastasis (59, 60). Hao et al. demonstrated that the combination of the fatty acid amide hydrolase (FAAH) inhibitor URB597 with the ferroptosis inducer RSL3 effectively modulates the PI3K-AKT signaling pathway, enhancing RCC cell sensitivity to ferroptosis and significantly inhibiting tumor growth and metastasis (61). Furthermore, dipeptidyl peptidase 9 (DPP9) competes with NRF2 for binding to KEAP1 in an enzyme-independent manner, thereby disrupting the KEAP1-NRF2 signaling pathway and influencing tumorigenesis and drug resistance in clear cell renal carcinoma (ccRCC) (11). The interaction between ferroptosis and tumor suppressor genes during tumor development serves as a natural defense mechanism (62). Conversely, ferroptosis escape mechanisms mediated by oncogenes or specific signaling pathways contribute to tumor initiation, progression, metastasis, and drug resistance (63–65). As a crucial tumor suppressor gene, P53 plays a key role in regulating the cell cycle, DNA repair, apoptosis, and metabolic pathways (66). In bladder cancer, P53 inhibits the activity of SLC7A11 (62), a critical component of System Xc- on the cell membrane, which is closely associated with ferroptosis (25). Inhibition of SLC7A11 activates ALOX15B lipoxygenase, which subsequently induces ferroptosis in BC cells (62).

**Table 1:** The mechanism of ferroptosis in urologic neoplasms.

Type of cancer	Biomolecular mechanism	Ref
Prostatic cancer (PC)	Dysregulation of lipid metabolism caused by ACSM1/3 deficiency leads to mitochondrial oxidative stress, lipid peroxidation and ferroptosis.	(76)
PC	CYLD promotes the expression of ACSL4 and TFRC through Hippo/YAP signaling and induces ferroptosis.	(77)
PC	The steroid saponin PPI promotes ROS and Fe2 + production through the ERK/DNMT1/ACSL4 axis, induces ferroptosis and inhibits cell proliferation.	(78)
PC	Antimony exposure inhibits ferroptosis by activating the Nrf2-SLC7A11-GPX4 pathway and promoting GPX4 expression.	(79)
PC	Androgen receptor mutation can induce up-regulation of SLC7A11 and inhibit ferroptosis induced by antiandrogen therapy.	(80)
PC	ASCL1 overexpression mediates CREB1 phosphorylation and promotes ferroptosis resistance in cells.	(81)
PC	RB1 loss/E2F activation increases the sensitivity of cancer cells to ferroptosis by up-regulating ACSL4 expression and PUFAs enrichment.	(82)
Renal carcinoma (RCC)	DPP9 upregulation mediates the stability of NRF2, promotes the expression of SLC7A11 and inhibits ferroptosis.	(11)
RCC	IL6 can reverse the SLC7A11 knockout effect through the JAK2/STAT3 pathway, inhibit ferroptosis and promote tumor resistance.	(83)
RCC	Knockout of SETD2 promotes the production of ROS and Fe^2 +^, and increases the sensitivity of cells to ferroptosis.	(84)
RCC	SMARCB1 regulates TFCP2L1-MYC transcription switch and inhibits ferroptosis.	(85)
RCC	Overcoming the compensatory increase of NRF2 induced by NPL4 inhibition enhances disulfiram/copper-induced oxidative stress and ferroptosis in RCC.	(86)
RCC	AIM2 promotes RCC progression and sunitinib resistance by regulating ferroptosis through FOXO3a/ACSL4 axis.	(73)
RCC	PDIA4 inhibits ferroptosis in renal cell carcinoma through the PERK/ATF4/SLC7A11 signaling pathway.	(87)
Bladder cancer (BC)	EGR1-regulated ALOX5 deficiency can promote ferroptosis resistance in bladder cancer.	(88)
BC	Silencing TFRC can inhibit ferroptosis in T24 cells.	(89)
BC	Brusatol induces ferroptosis by reducing the expression of SLC7A11 and NRF2 through the Chac1/Nrf2/SLC7A11 pathway.	(90)
BC	The endogenous peptide CTSGDP-13 promotes ferroptosis in BC by regulating the USP7/TRIM25/KEAP1 axis.	(91)
BC	TalaA promotes ferroptosis by increasing intracellular reactive oxygen species and up-regulating transferrin and heme oxygenase 1, thereby inhibiting tumor cell proliferation, DNA replication and colony formation.	(92)
BC	PCBP1 enhances mitochondrial function and reduces ROS production by inducing LACTB mRNA degradation, thereby inhibiting erastin-mediated ferroptosis.	(93)

It is noteworthy that certain tumor cells exhibit intrinsic sensitivity to ferroptosis due to their unique metabolic characteristics, elevated levels of ROS, and specific gene mutations. This sensitivity reveals potential therapeutic vulnerabilities in particular cancer types (67, 68). Therefore, ferroptosis is recognized as one of the key mechanisms of cell death induced by various cancer therapies, including radiotherapy, immunotherapy, chemotherapy, and targeted therapies (69–72). Multiple studies have demonstrated that ferroptosis can enhance the sensitivity of urinary system cancers to chemotherapy through the modulation of associated pathways (73–75). For instance, Wang et al. found that absent in melanoma 2 (AIM2) can promote the progression of renal cell carcinoma and sunitinib resistance independently of the inflammasome (73). Mechanistic studies revealed that AIM2 facilitates the phosphorylation and proteasomal degradation of FOXO3a, subsequently inhibiting the transcriptional activity of ACSL4. This inhibition of ACSL4 contributes to ferroptosis resistance and sunitinib resistance in kidney cancer. Interestingly, the ferroptosis inducer RSL3 demonstrated a synergistic effect with sunitinib, suggesting that targeting ferroptosis may represent a novel approach for treating sunitinib-resistant kidney cancer (73). Additionally, docetaxel resistance in prostate cancer and cisplatin resistance in bladder cancer are also closely linked to ferroptosis (74, 75). Therefore, an in-depth exploration of the molecular mechanisms underlying ferroptosis and its intricate regulatory network is essential for developing effective treatment strategies for malignant urinary system tumors.

## 4 Non-coding RNA targeted regulation of ferroptosis

Non-coding RNA (ncRNA), a distinct class of RNA molecules that do not directly encode proteins, plays a crucial role in biological processes at the RNA level and is extensively involved in regulating gene expression (94). Among these ncRNAs, microRNA (miRNA), circular RNA (circRNA), and long non-coding RNA (lncRNA) are key regulatory molecules currently under extensive investigation. These molecules influence gene expression through various mechanisms, including transcriptional regulation, post-transcriptional regulation, and epigenetic modifications (95).

MiRNAs are a class of non-coding, single-stranded RNA molecules approximately 21-23 nucleotides in length, encoded by endogenous genes (96, 97). They induce cleavage, degradation, or translation inhibition of target mRNA through complementary binding to the 3’-UTR region of the target mRNA, thereby inhibiting the expression of target genes (97, 98). Recent studies have demonstrated that various miRNAs can regulate ferroptosis by targeting ferroptosis-related genes. For instance, miR-545 suppresses its expression by binding to TF mRNA, which reduces iron uptake and subsequently inhibits ferroptosis while promoting CRC cell survival (99). Conversely, miR-30b-5p and miR-124 diminish iron excretion by inhibiting the expression of FPN1 (an iron export protein), thereby promoting ferroptosis (100, 101). LncRNAs are a class of non-coding RNA molecules that exceed 200 nucleotides in length (102). They regulate gene expression at the epigenetic level through various mechanisms, including chromatin remodeling, transcriptional regulation, translation regulation, and post-translational modification (103). For instance, Wang et al. found that lncRNA-LINC00618 reduces SLC7A11 transcription in leukemia, thereby regulating ferroptosis at the transcriptional level (104). Additionally, lncRNAs can function as competitive endogenous RNAs (ceRNAs). They act as “sponges” for microRNAs (miRNAs), absorbing miRNAs and reducing their inhibitory effects on target mRNAs, thereby indirectly upregulating target gene expression (105, 106). For example, lncRNA-NEAT1 enhances MIOX expression by competitively binding to miR-362-3p, thereby increasing ferroptosis and offering a potential strategy for improving chemotherapy sensitivity in hepatocellular carcinoma (HCC) patients (107). CircRNAs are another class of non-coding RNA molecules characterized by a closed ring structure, lacking a 5’-terminal cap and a 3’-terminal poly(A) tail (108, 109). Current studies indicate that circRNAs primarily regulate ferroptosis by modulating downstream gene expression as miRNA sponges. Wang et al. reported that circ_0067934 attenuates ferroptosis and promotes cell proliferation by targeting the miR-545-3p/SLC7A11 signaling pathway in thyroid cancer cells (110). CircIL4R binds to miR-541-3p, inhibiting miR-541-3p and enhancing GPX4, thereby suppressing ferroptosis in hepatocellular carcinoma (111). Numerous studies have confirmed that circRNAs can function as ceRNAs to regulate ferroptosis (112–114).

Existing studies have demonstrated that ncRNAs regulate ferroptosis by targeting metabolic mechanisms associated with this process, with recent reviews summarizing this area of research (115–118). Overall, ferroptosis is influenced by iron metabolism, lipid metabolism, and amino acid metabolism. Numerous proteins, particularly transcription factors, modulate ferroptosis either directly or indirectly through the regulation of these metabolic pathways. For instance, in the iron metabolism pathway, iron uptake is mediated through interactions between transferrin (TF) and transferrin receptor (TFRC), as well as the reduction of STEAP3. Ferritin, heat shock protein β-1 (HSPB1), and iron response element binding protein 2 (IREB2) facilitate iron utilization and export via iron transporter 1 (FPN, also known as SLC40A1) (119–123). Overall, ferroptosis is influenced by iron metabolism, lipid metabolism, and amino acid metabolism. Numerous proteins, particularly transcription factors, modulate ferroptosis either directly or indirectly through the regulation of these metabolic pathways. For instance, in the iron metabolism pathway, iron uptake is mediated through interactions between transferrin (TF) and transferrin receptor (TFRC), as well as the reduction of STEAP3. Ferritin, heat shock protein β-1 (HSPB1), and iron response element binding protein 2 (IREB2) facilitate iron utilization and export via iron transporter 1 (FPN, also known as SLC40A1) (42, 44, 45, 124, 125). NcRNAs primarily regulate ferroptosis through interactions with key enzymes involved in these enzymatic reactions. Amino acid metabolism is critically dependent on the stable function of System Xc-. SLC7A11 and SLC3A2 are crucial components of System Xc-, responsible for transporting glutathione (GSH). GPX4 utilizes GSH as a reducing agent and serves as a key protective enzyme in the antioxidant defense system (36). Currently, most ncRNAs predominantly target SLC7A11 and GPX4 mRNA to modulate amino acid metabolism, thereby influencing ferroptosis. Additionally, ncRNAs can affect other regulators of ferroptosis, such as NRF2, p53, and FSP1 (126–128).

In conclusion, ncRNAs possess significant potential in modulating ferroptosis, with mechanisms of action that are both intricate and varied. As research progresses, it is anticipated that the detailed roles and mechanisms of ncRNAs in ferroptosis will be elucidated, potentially leading to novel strategies for the prevention and treatment of related diseases. **Table 2** presents information regarding the regulation of ferroptosis by ncRNAs, thereby enhancing our understanding of the research advancements in this domain.

**Table 2:** Regulation of ferroptosis by non-coding RNAs.

NcRNA	Target	Effect on ferroptosis	Function	Ref
miR-3648	SOCS2	Promote	Inhibition of non-small cell lung cancer (NSCLC) proliferation, migration and invasion	(129)
miR-26a-1-3p	MDM2	Promote	Induced ROS accumulation, GSH consumption, mitochondrial contraction and lipid peroxidation.	(130)
miR-139	NRF2	Promote	Increased radiosensitivity of NSCLC cells *in vitro* and *in vivo*.	(131)
miR-335-5p	GPX4	Inhibit	Inhibition of ferroptosis promotes breast cancer progression	(132)
miR-214-3p	ACSL4	Inhibit	Reduce the anti-angiogenesis effect mediated by apatinib	(133)
miR-152	TFR1	Inhibit	Inhibit proliferation in HCC	(134)
LncASMTL-AS1	SAT1	Promote	Inhibit cell growth	(135)
LncHCP5	HCP-132aa	Promote	Promote cell growth	(136)
LINC00618	SLC7A11	Promote	Promote apoptosis and sensitivity to vincristine	(104)
LncRNA-NRAV	miR-375-3P/SLC7A11	Inhibit	Enhance iron output and increase ferroptosis resistance	(137)
LINC02086	miR-342-3p/CA9	Inhibit	Promote the proliferation, migration and invasion of PC cells.	(138)
LncRNA H19	miR-19–3p/ FTH1	Inhibit	Inhibit sensitivity to curcumenol	(139)
CircFOXP1	OTUD4	Promote	Regulating the stability of NCOA4 protein to enhance ferroptosis of ICC	(140)
CircSCN8A	miR-1290/ ACSL4	Promote	Inhibit NSCLC proliferation, migration, invasion, and epithelial-mesenchymal transition	(141)
CircBCAR3	miR-27a-3p/ TNPO1	Promote	Promote proliferation, migration, invasion	(142)
CircDTL	miR-1287-5p/ GPX4	Inhibit	Promote cell growth, inhibit apoptosis and sensitivity to chemotherapeutic	(143)
CircBBS9	miR-7150	Inhibit	Regulate ferroptosis and immune microenvironment in lung adenocarcinoma	(144)
CircPIAS1	miR-455-3p/ NUPR1	Inhibit	Promote the proliferation and migration of HCC cells.	(145)

## 5 Non-coding RNAs target ferroptosis in urologic cancers

### 5.1 MiRNA-ferroptosis axis in urologic tumors

In the complex pathophysiological mechanisms underlying urinary system cancers, the interplay between miRNAs and ferroptosis has increasingly emerged as a focal point of scientific investigation. MiRNAs, a class of short, non-coding RNAs, play a significant role in regulating cellular physiological and pathological processes through the precise modulation of target gene expression (146). Ferroptosis, an emerging mode of cell death, is characterized by the regulation of intracellular iron metabolism and lipid peroxidation; its role in cancer has been progressively elucidated. In the context of urinary system cancers, the relationship between miRNAs and ferroptosis is particularly noteworthy. Studies have demonstrated that several miRNAs act as key regulators of ferroptosis-related genes, thereby influencing the survival and proliferation of cancer cells by modulating the ferroptosis process. For example, Huang et al. identified that miR-217, derived from bladder cancer (BC) tissue exosomes, plays a crucial role in inhibiting ferroptosis in BC. Functionally, the introduction of miR-217 into cells resulted in a reduction of malondialdehyde (MDA), lactate dehydrogenase (LDH), and Fe²^+^ levels, while simultaneously increasing the level of superoxide dismutase (SOD), thus significantly inhibiting both ferroptosis and apoptosis (147). This finding elucidates the potential mechanisms by which miRNAs regulate ferroptosis in urinary system tumors.

Further exploration reveals that the mechanistic role of the miRNA-ferroptosis axis in urinary system tumors operates at multiple levels. On one hand, miRNAs can directly target crucial genes in the ferroptosis pathway, such as ferritin heavy chain 1 (FTH1) and solute carrier family 40 member 1 (SLC40A1), which are essential for maintaining intracellular redox balance and iron homeostasis (148, 149). The significant role of NRF2 and its target genes in preventing ferroptosis positions this pathway as a promising target for cancer treatment. The NRF2/FTH1 axis is critical for maintaining cellular iron metabolism balance (150). In prostate cancer, miR-29a-5p modulates ferroptosis by influencing the levels and accumulation of intracellular Fe^2+^ and MDA via the NRF2/FTH1 axis (148). Knocking down or inhibiting miR-29a-5p can activate ferroptosis, thereby suppressing the proliferation of PC cells both *in vitro* and *in vivo*, providing new insights for subsequent cancer treatment (148). Similarly, miR-4735-3p impedes the growth of clear ccRCC cells by binding to the 3’-UTR of SLC40A1 and downregulating its expression, which resultsing in iron overload and ferroptosis (149). Conversely, miRNAs can mediate interactions between tumor cells and macrophages, thereby indirectly regulating ferroptosis (151). Xiao et al. found that tumor-associated macrophages secrete taurine to inhibit ferroptosis by activating the LXRα/SCD1 axis in PC. Mechanistically, LXRα promotes ferroptosis through its downstream target, stearoyl-CoA desaturase 1 (SCD1), which is recognized as a ferroptosis suppressor gene; its overexpression inhibits ferroptosis. Concurrently, LXRα can upregulate the expression of miR-181a-5p and the RNA-binding protein FUS, promoting the enrichment of miR-181a-5p in tumor-derived exosomes. These miR-181a-5p-enriched exosomes can subsequently modulate macrophage M2 polarization and upregulate the expression of the taurine transporter gene TauT by targeting the Hippo-YAP pathway. This process increases taurine secretion and further inhibits ferroptosis in PC. In summary, the study by Xiao et al. demonstrates that miR-181a-5p can shuttle from tumor cells to macrophages, enhancing taurine secretion as a feedback mechanism and thereby facilitating crosstalk between macrophages and tumor cells (151). This finding provides a novel perspective on the role of miRNAs in mediating interactions between tumor cells and immune cells.

The miRNA-ferroptosis axis demonstrates significant potential in the treatment of urinary system tumors. Acquired chemotherapy resistance frequently occurs in advanced tumors, complicating treatment efforts. Since ferroptosis acts as a tumor suppressor in cancer development, its activation may represent an effective anticancer strategy (152). Research has shown that cancer-associated fibroblast (CAF)-derived exosomes can impede ROS accumulation in prostate cancer cells and reduce mitochondrial damage, thereby inhibiting iron-induced ferroptosis in these cells and promoting acquired chemotherapy resistance (153). Zhao et al. found that miR-432-5p within CAF-derived exosomes inhibits the ferroptosis process by targeting CHAC1 expression, thereby enhancing the resistance of prostate cancer cells to docetaxel. Notably, the knockdown of miR-432-5p expression in CAFs significantly increases tumor sensitivity to chemotherapy (153). Consequently, a comprehensive investigation of the miR-432-5p/CHAC1 axis may provide a promising strategy to counteract acquired chemotherapy resistance in advanced tumors. Moreover, certain anti-tumor compounds have been shown to impact the miRNA-ferroptosis axis. Icariin II (ICS II), an active flavonoid derived from the traditional Chinese medicine Epimedium, exhibits anti-tumor activity across various cancers (154, 155). Yu et al. demonstrated that ICS II inhibits the proliferation, migration, and invasion of RCC cells (156). Functionally, ICS II promotes the accumulation of Fe^2+^, MDA, and ROS in RCC cells while reducing glutathione (GSH) levels, thereby inducing ferroptosis. Mechanistically, ICS II downregulates GPX4 expression through miR-324-3p, and this pathway operates independently of p53. Consequently, ICS II may serve as a promising therapeutic agent for RCC by inducing ferroptosis in RCC cells via its action on the miR-324-3p/GPX4 axis (156). Further research has indicated that the combined application of icariin (ICA) and curcumin produces a synergistic effect in inducing apoptosis and ferroptosis, significantly inhibiting the viability and proliferation of PC cells (157). Both compounds affect lipid metabolism in PC by modulating the miR-7/mTOR/SREBP1 pathway, thereby influencing autophagy and ferroptosis (157).

**Figure 2:**
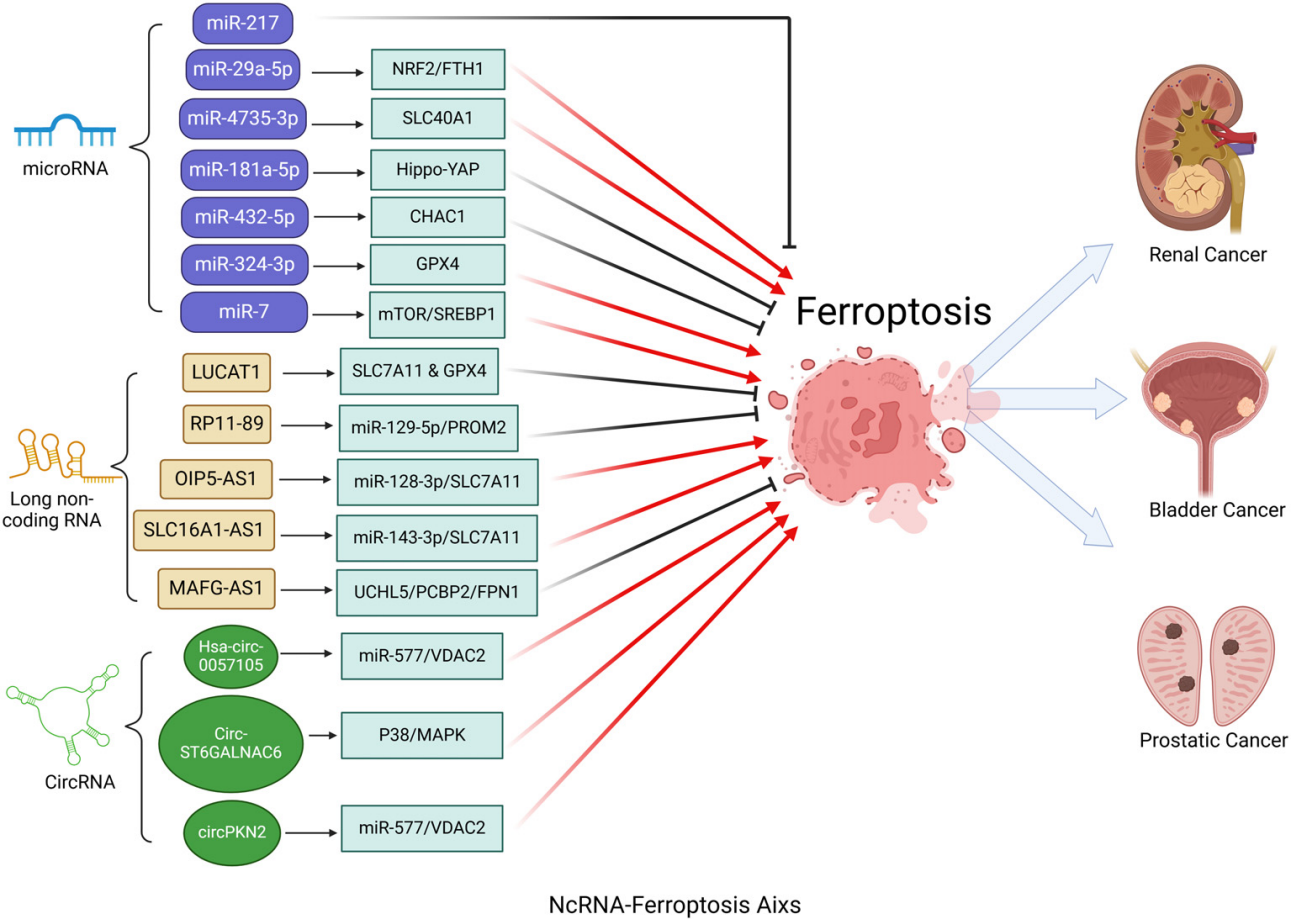
Critical role of the ncRNA-ferroptosis axis in urologic cancers. MicroRNAs (miRNAs) influence ferroptosis in urologic cancers by regulating genes associated with ferroptosis. Long non-coding RNAs (lncRNAs) and circular RNAs (circRNAs) regulate ferroptosis primarily by targeting specific microRNAs (miRNAs) through a sponge effect.

In the progression of urinary system tumors, miRNAs modulate ferroptosis by targeting ferroptosis-related genes, thereby influencing tumor cell proliferation, invasion, and immune modulation (**Figure 2**). The aforementioned studies have identified promising avenues for the development of novel ferroptosis-regulating targets, including the miRNA-217, miR-29a-5p/NRF2/FTH1 axis, miR-4735-3p/SLC40A1 axis, and the miR-181a-5p/Hippo-YAP signaling pathway. These targets have the potential to impede the progression of urinary system tumors. In the realm of cancer therapy, precise control of ferroptosis through pathways such as miR-432-5p/CHAC1, miR-324-3p/GPX4, and miR-7/mTOR/SREBP1 presents a promising strategy to enhance the efficacy of drug treatments. This approach not only supports ongoing research into therapeutic interventions but also lays the groundwork for the development of innovative anticancer therapies. Consequently, there is an urgent need to further explore the crucial role of the miRNA-ferroptosis axis in urinary system cancers, as it may enrich our understanding of tumorigenesis and unlock the potential to transform cancer treatment, ultimately enhancing patient outcomes.

### 5.2 LncRNA-ferroptosis axis in urologic tumors

Given the ability of long non-coding RNAs (lncRNAs) to regulate gene expression at multiple levels—including chromatin remodeling, transcription, translation, and post-translational regulation—the lncRNA-ferroptosis axis in urinary system cancers has been the subject of extensive research (103). Primarily, lncRNAs regulate ferroptosis in urinary system tumors through gene transcription. For instance, the high expression of lncRNA-LUCAT1 significantly inhibits ferroptosis in bladder cancer (BC) (158). Cao et al. reported that LUCAT1 binds to the m6A reader IGF2BP1 to form a LUCAT1-IGF2BP1 complex, which stabilizes STAT3 mRNA and inhibits the expression of ferroptosis-related genes, including down-regulation of COX2, ACSL4, and NOX1, as well as up-regulation of SLC7A11 and GPX4. This mechanism promotes the migration and invasion of BC cells (158). Additionally, lncRNA MAFG-AS1 also inhibits ferroptosis in bladder cancer by modulating iron metabolism-related proteins (159). Further investigations have shown that high expression of MAFG-AS1 correlates with poor prognosis in bladder cancer patients, while those with low expression exhibit greater sensitivity to adjuvant chemotherapy involving cisplatin and gemcitabine. In conclusion, this study highlights the critical role of the MAFG-AS1/UCHL5/PCBP2/FPN1 axis in cisplatin resistance in bladder cancer, suggesting that inhibition of MAFG-AS1 may promote ferroptosis and enhance cellular sensitivity to cisplatin treatment (159). Second, lncRNA functions as a ceRNA that regulates ferroptosis in urinary system cancers (160–162). LncRNA can exert a “sponge effect” on miRNA, thereby promoting the upregulation of related target gene expression. An illustrative example is lncRNA RP11-89 in BC, which upregulates the expression of PROM2 by sponging miR-129-5p, consequently activating iron output and inhibiting ferroptosis in BC. This finding elucidates the novel role of RP11-89 as an oncogene and suggests the potential for treating BC by modulating the miR-129-5p/PROM2 axis (160). Similarly, lncRNA OIP5-AS1 and SLC16A1-AS1 regulate ferroptosis through the miR-128-3p/SLC7A11 and miR-143-3p/SLC7A11 signaling axes in prostate cancer and kidney cancer, respectively, further confirming the extensive role of lncRNA in regulating ferroptosis in urinary system cancers (161, 162).

As research on urinary system cancers deepens, an expanding body of evidence indicates that ncRNAs, particularly ferroptosis-associated lncRNAs (FRLs), play a significant role in the occurrence, development, and prognosis of these malignancies. Bioinformatics analyses further underscore the considerable potential of FRLs as prognostic markers for urological cancers.

In 2021, Xing et al. first identified that three FRLs—DUXAP8, LUCAT1, and LINC02609—were significantly associated with the overall survival (OS) of renal clear cell carcinoma (163). The risk assessment model developed using these FRLs can accurately predict ccRCC prognosis and offers novel approaches for cancer prognosis screening. This finding not only enhances our understanding of ccRCC prognosis but also provides a critical foundation for the development of personalized treatment options. In a subsequent study, Lai et al. integrated data from the TCGA and FerrDb databases, screening a total of 433 FRLs. They conducted univariate Cox regression, Lasso regression, and multivariate Cox regression analyses, identifying eight FALs (LINC00460, AC124854.1, AC084876.1, IGFL2-AS1, LINC00551, AC083967.1, AC073487.1, and LINC02446) as prognostic features of ccRCC (164). They developed a risk assessment model, FLPS, using these eight FALs. This model not only demonstrates independent predictive value for ccRCC but also elucidates how lncRNAs involved in FLPS influence the progression and survival outcomes of ccRCC. Furthermore, they found that FLPS was closely related to immune infiltration, suggesting the potential therapeutic value of immune checkpoint inhibitors for high-risk ccRCC patients. This discovery provides a new strategy and direction for immunotherapy in ccRCC (164). In renal papillary cell carcinoma (KIRP), Wu et al.’s study also revealed the potential of FRLs as biomarkers (165). They identified a specific group of FRLs associated with both the occurrence and progression of KIRP. CASC19, AC090197.1, AC099850.3, AL033397.2, LINC00462, and B3GALT1-AS1 were significantly upregulated in high-risk groups, suggesting their potential role as oncogenic factors. Conversely, LNCTAM34A and AC024022.1 were significantly upregulated in the low-risk group, indicating their potential function as tumor suppressor factors in KIRP. The differential expression patterns of these FRLs in high-risk and low-risk groups imply that they may serve as either tumor-promoting or tumor-suppressing factors. These findings provide a crucial reference for the early diagnosis, prognosis evaluation, and treatment selection of KIRP.

Studies have demonstrated that a variety of functional RNA molecules (FRLs) serve as prognostic markers not only in renal cell carcinoma but also in prostate and bladder cancers (166–168). FRLs influence tumor cell proliferation and apoptosis directly, while also modulating immune cell infiltration and function by regulating the tumor immune microenvironment. This dual role contributes significantly to tumor development and progression. Therefore, the critical importance of FRLs in urinary system cancers must be acknowledged. Identifying FRLs associated with urinary system tumors and developing a prognostic risk assessment model based on these FRLs will facilitate more accurate prognosis predictions and enable the creation of personalized treatment and monitoring plans. This approach not only enhances the relevance and effectiveness of cancer treatment but also improves patients’ quality of life and extends their survival.

The aforementioned studies have illuminated two critical aspects of the lncRNA-ferroptosis axis in urinary system tumors. Firstly, lncRNAs significantly influence tumor progression and treatment response by modulating ferroptosis. Specifically, lncRNAs such as LUCAT1, MAFG-AS1, RP11-89, OIP5-AS1, and SLC16A1-AS1 exert their effects by regulating ferroptosis-related genes, thereby impacting the development and management of urinary system cancers. Notably, the role of lncRNAs as miRNA sponges adds an additional layer of complexity to this regulatory network, underscoring the necessity for a comprehensive understanding of their interactions. Elucidating the precise mechanisms by which these lncRNA-mediated ferroptotic processes contribute to tumorigenesis is essential for enhancing our understanding of cancer development. Secondly, FRLs identified through bioinformatic methods underscore their strong association with patient prognosis. With an anticipated continuous increase in the identification of FRLs, the establishment of more accurate and sensitive prognostic models based on these biomarkers represents a significant advancement for urinary system cancers.

### 5.3 CircRNA-ferroptosis aixs in urologic tumors

Similar to lncRNAs, circRNAs can regulate the expression of downstream genes by acting as miRNA sponges, thereby influencing the process of ferroptosis in tumors. Although relatively few studies have focused on circRNAs in urinary system cancers, existing research has highlighted their significant potential in this area. Cen et al. demonstrated that circRNA (Has-circ-0057105) functions as a sponge for miR-577 in renal cell carcinoma, modulating the expression of the COL1A1 and VDAC2 genes at both mRNA and protein levels (169). VDAC2 is a nuclear-encoded mitochondrial protein involved in ferroptosis, responsible for regulating the exchange of ions and metabolites between the cytosol and mitochondria (170). Notably, Overexpression of VDAC2 is closely associated with sensitivity to ferroptosis (40, 170). VDAC2 overexpression did not directly induce ferroptosis in their experiments, nor did it influence cell proliferation or levels of intercellular iron, MDA, and GSH. However, when the ferroptosis inducer Erastin was administered to RCC cells, a more pronounced ferroptosis effect was observed in the VDAC2 overexpression group, characterized by iron accumulation, MDA overproduction, and GSH depletion (169). In summary, this study illustrates that Hsa-circ-0057105 regulates ferroptosis sensitivity in RCC through the miR-577/VDAC2 axis, providing a novel target for RCC treatment. Additionally, circRNAs can bind to functional proteins, influence downstream signaling pathways, and regulate ferroptosis. Wang et al. elucidated the mechanism by which circST6GALNAC6 regulates ferroptosis in BC (171). CircST6GALNAC6 interacts with HSBP1, inhibiting its phosphorylation at serine-15, and thereby activating the P38/MAPK signaling pathway to promote ferroptosis (171). Recently, a novel circRNA, circPKN2, has been identified as significantly down-regulated in bladder cancer and closely associated with patient prognosis (172). Subsequent studies have demonstrated that circPKN2 promotes the ubiquitination of SCD1 and inhibits the WNT pathway by recruiting STUB1 in bladder cancer, thereby enhancing ferroptosis in BC cells and inhibiting tumor growth and metastasis (172).

Therefore, the circRNA-ferroptosis axis plays a crucial role in the development of urinary system cancers (**Figure 2**). A detailed investigation into the specific mechanisms of these circRNAs in ferroptosis may pave the way for novel therapeutic strategies in the treatment of urinary system cancers.

## 6 Limitations and future perspectives

To the best of our knowledge, this is a review that summarizes the evidence connecting ncRNAs and ferroptosis in urological oncology. As illustrated in **Figure 2**, ncRNA-mediated ferroptosis may play a crucial role in the malignant transformation of urinary system cancers, significantly impacting cancer cell proliferation, cell cycle progression, migration, invasion, and angiogenesis. Regarding the miRNA-ferroptosis axis, ferroptosis mediated by miR-27, miR-29a-5p, miR-181a-5p, and miR-4735-3p regulates urinary system tumors by influencing cancer cell proliferation. Concurrently, ferroptosis mediated by miR-432-5p, miR-7, and miR-324-3p contributes to cancer therapy by altering tumor sensitivity to drug treatments. However, to date, no miRNA-ferroptosis axis specifically targeting bladder cancer treatment has been identified. For the lncRNA-ferroptosis axis, lncRNAs such as LUCAT1 and MAFG-AS1 directly target ferroptosis-related genes to regulate ferroptosis, thereby influencing bladder cancer development and drug resistance. Conversely, lncRNAs RP11-89, OIP5-AS1, and SLC16A1-AS1 function as miRNA sponges, modulating ferroptosis in the urinary system by targeting corresponding miRNAs. Notably, numerous FRLs have been identified through bioinformatic analyses, demonstrating potential as independent prognostic factors. Prognostic risk models based on these FRLs hold significant clinical value, assisting physicians in accurately assessing patient conditions and formulating personalized treatment plans. Although research on the circRNA-ferroptosis axis remains limited, studies have revealed that ferroptosis mediated by Hsa_circ_0057105, CircST6GALNAC6, and circPKN2 plays important roles in renal and bladder cancers. Future investigations into the circRNA-ferroptosis axis in prostate cancer are anticipated. In light of these findings, targeting ncRNAs for the modulation of ferroptosis may emerge as reliable diagnostic and prognostic biomarkers, as well as promising therapeutic strategies in urological oncology.

When interpreting this comprehensive review, it is essential to acknowledge certain inherent limitations. First, due to the scarcity of clinical studies, further research is necessary to thoroughly elucidate the diagnostic, therapeutic, and prognostic value of ncRNAs and ferroptosis in urinary system cancers. Second, the molecular mechanisms underlying ncRNA-mediated ferroptosis and urinary system tumorigenesis require further clarification and investigation. In addition to targeting genes and corresponding signaling pathways, other factors, such as the tumor microenvironment and tumor immunity, may also play critical roles in the pathogenesis and progression of urinary system cancers. Finally, it is important to note that ncRNAs exhibit dual roles in cancer, acting as both tumor suppressors and promoters in specific urinary system tumors. Consequently, future studies should focus on exploring the specific mechanisms of ncRNAs within ferroptosis signaling pathways, particularly their interactions with vital biological processes such as iron metabolism, lipid peroxidation, and the antioxidant system. By unraveling these interactions, we can gain a deeper understanding of the biological functions of ncRNAs in the progression of urinary system cancers, including cell proliferation, epithelial-mesenchymal transition, and the induction of chemotherapy resistance. Based on the regulatory mechanisms of the ncRNA-ferroptosis axis, novel ferroptosis-inducing therapies are anticipated in the future.

## 7 Conclusion

This review highlights that ncRNA-mediated ferroptosis is a significant molecular mechanism involved in the onset and progression of urinary system tumors, such as prostate cancer, bladder cancer, and renal cancer. The regulation of ncRNAs, whether through upregulation or downregulation, can either activate or inhibit ferroptosis in these malignancies. By precisely modulating the expression of genes related to ferroptosis, ncRNAs are instrumental in this process, as they influence the metabolic balance of intracellular iron ions, lipid peroxidation reactions, and antioxidant defense systems.
